# Nebulised heparin as a treatment for lung diseases: formulation challenges and pulmonary drug delivery strategies

**DOI:** 10.1007/s00408-026-00905-y

**Published:** 2026-07-01

**Authors:** Margarida Miranda, Marc Brown, Frank M. P. van Haren, Beth Brown, Clive P. Page

**Affiliations:** 1 MLBT Investments and Consultancy, 2 Crossways Business Centre, Bicester Road, Kingswood, Aylesbury, UK; 2 Coimbra Chemistry Center, Department of Chemistry, University of Coimbra, Coimbra, Portugal; 3 Egas Moniz School of Health & Science, Egas Moniz Center for Interdisciplinary Research (CiiEM), Caparica, Almada, 2829-511 Portugal; 4 St George Hospital, Sydney, Australia; 5 College of Science and Medicine, Australian National University, Canberra, Australia; 6 Pulmonary Pharmacology Unit, Institute of Pharmaceutical Science, King’s College London, London, UK

**Keywords:** Heparin, Anti-inflammatory, Pulmonary, Drug delivery, Anti-infective, Formulation development.

## Abstract

The COVID-19 pandemic further emphasized the global demand for heparin and its expanding clinical relevance, indicating that even one of the oldest drugs in medicine continues to reveal new therapeutic horizons. Traditionally recognized for its anticoagulant and antithrombotic activities, heparin is increasingly being explored for its versatile therapeutic potential in the treatment of a range of pulmonary diseases, including respiratory infections (e.g. COVID-19), Acute Respiratory Distress Syndrome (ARDS), asthma, chronic obstructive pulmonary disease (COPD) and cystic fibrosis. In all of these diseases, inhaled unfractionated heparin (UFH) therapy has been investigated in a number of clinical trials that have demonstrated promise for this drug when administered directly to the lungs. However, using heparin by this “off label” route of administration, poses a number of technical challenges: the physicochemical properties of heparin at therapeutic doses often results in highly viscous formulations, causing device blockage and drug sorption during nebulization. These limitations underscore the need for innovative formulation strategies to improve aerosol flow, reduce dosing inefficiencies, and enable reliable pulmonary administration. Advancing heparin formulations for delivery to the lung could therefore unlock significant benefits for a wide spectrum of respiratory disorders, marking a new chapter in the long medical history of this drug as discussed below.

## Introduction

Heparin, a naturally occurring glycosaminoglycan, has been recognized for its anticoagulant properties for more than a century. Its discovery in 1916 marked the beginning of its extensive use in various medical fields traditionally being used for its anti-coagulant and antithrombotic activity, primarily through its ability to enhance the activity of antithrombin, a key inhibitor in the coagulation cascade [1]. However, emerging evidence has illuminated a broader spectrum of biological activities associated with heparin, particularly its anti-inflammatory effects and pan-anti-infective actions [2–4]. This increased understanding of the broader pharmacology of heparin has sparked interest in the use of the drug, beyond an anticoagulant [5].

The pathophysiology of inflammation is complex, involving a cascade of cellular and molecular events that lead to the recruitment of immune cells and the release of pro-inflammatory mediators, from a range of inflammatory cells. In many chronic inflammatory diseases, the dysregulated inflammatory response contributes to tissue damage and disease progression. Many studies have now demonstrated that heparin can modulate inflammatory responses by modulating various cellular and molecular pathways [6–9]. Heparin has a number of direct anti-inflammatory effects; for instance, heparin has been reported to inhibit the action of neutrophil elastase, a protease that plays a pivotal role in tissue damage during inflammatory responses in the lung in diseases such as COPD, cystic fibrosis and bronchiectasis [1, 10]. Furthermore, its ability to disrupt neutrophil extracellular traps (NETs) may provide an additional mechanism through which heparin can attenuate inflammation and prevent further tissue injury [1, 8]. This anti-inflammatory action may contribute to heparin’s efficacy in treating diseases characterized by excessive neutrophilic inflammation, such as ARDS and other chronic inflammatory disorders of the lung such as COPD and cystic fibrosis [11].

The relevance of heparin’s broad range of pharmacological properties has gained particular prominence in the context of the recent COVID-19 pandemic [12]. Observations of inflammation and thrombotic events in the lung as a consequence of SARS-CoV-2 infection led to the use of nebulised heparin in the treatment of COVID-19 with a recent meta trial conducted across six countries demonstrating a highly significant reduction in mortality and in reducing the need for ventilation [13]. This positive series of clinical trials adds to the more than 50 other clinical studies successfully using inhaled heparin in a range of respiratory diseases, reviewed in [2, 14] which evaluated both the safety and efficacy of this approach. Surprisingly for a drug being used “off label” in so many positive clinical trials, there is currently no approved inhaled formulation of heparin which has led to we and others [15] now developing novel heparin formulations for inhalation and delivery methods as a novel approach for the treatment of a range of inflammatory conditions of the lung (discussed in more detail below).

This review aims to provide an overview of the current understanding of heparin’s anti-inflammatory mechanisms, its potential usefulness as a treatment for various inflammatory conditions in the lung, and the ongoing research efforts aimed at harnessing its therapeutic benefits. From a practical perspective, formulations and devices suitable for pulmonary delivery of heparin are also discussed, as this route of administration is constrained by multiple mechanical, chemical, physiological, and behavioural barriers, thereby requiring careful consideration during formulation development studies.

By elucidating the multifaceted roles of heparin, together with applicable formulation strategies, we hope to contribute to the growing body of literature that supports the use of inhaled heparin in the treatment of a range of inflammatory diseases of the lung.

## Heparin: structure, function and source

Heparin is a naturally occurring glycosaminoglycan predominantly synthesised and stored in mast cell granules, especially in mucosal tissues, such as the liver, gut and respiratory tract [11, 16]. Structurally, it is a highly sulphated form of heparan sulphate, another glycosaminoglycan, produced by most mammalian cells [1, 11, 17, 18]. While heparan sulphate typically exists covalently attached to core proteins as part of proteoglycans on cell surfaces and in the extracellular matrix, heparin is mainly stored intracellularly in mast cell granules, with only small amounts found in free form [11, 16, 18].

**Fig. 1:**
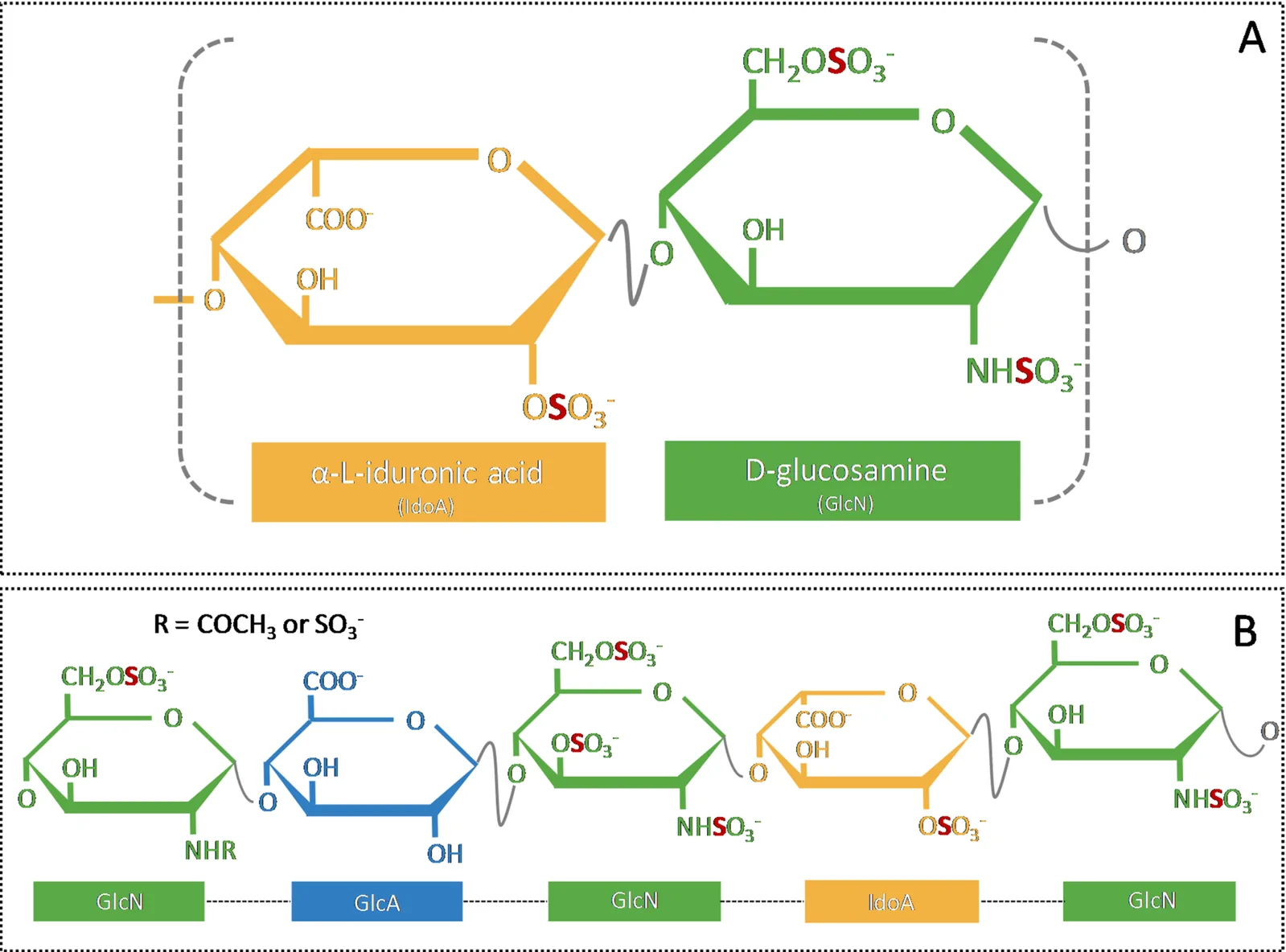
**A** Repeating sulphated disaccharide units composed of 1,4-linked α-L-iduronic acid and α-D-glucosamine; **B** Predominant pentasaccharide sequence responsible for anticoagulant effect in porcine intestinal heparin. Adapted from [1, 5].

In essence, heparin backbone consists of repeating sulphated disaccharide units of 1,4 linked α-L-iduronic (IdoA) or β-D-glucuronic acid (GlcA) and α-D-glucosamine (GlcN). However, these saccharide chains can be of distinct lengths, ranging from 10 to over 100 monosaccharide units [19]. Furthermore, not all the chains have the same composition, with several sulphation patterns being possible. All together, these factors contribute to a marked microheterogeneity in its structure. As highlighted in Fig. 1A, a trisulfated disaccharide GlcNS6S (1 → 4) IdoA2S is the major repeating unit in heparin [5, 19–21].

Heparin is mostly known for its anticoagulant effect which is mainly ascribed to a specific pentasaccharide sequence – GlcNAc/S6S → GlcA→GlcNS3S6S → IdoA2S → GlcNS6S (see Fig. 1B).

The recognition of this sequence by antithrombin activates a conformational change in the molecule leading to a rapid inhibition of thrombin (FIIa) and factor Xa, thus inhibiting the coagulation cascade. However, due to the heterogeneous structure of naturally occurring heparin, this unique pentasaccharide sequence is only found in about one third of the heparin chains [5, 11]. Furthermore, for a successful inhibition of thrombin – heparin, size matters. This means that for thrombin to be inhibited, a minimum of 18 saccharide units of heparin are required, as the drug needs to be simultaneously connected to both thrombin and antithrombin. However, this parameter is not as crucial for FXa inhibition, as this protease only requires the binding of the pentasaccharide sequence to antithrombin [11].

In mammals, heparin is only synthetized in mast cells, which are predominantly circumscribed to mucosal and connective tissues. Although heparin can be extracted from several animal sources (e.g. cattle, camels, sheep, goats and turkeys) most pharmaceutical heparin is currently produced from intestinal mucosa, mainly from Chinese pigs. Prior to 1990, bovine sources (primarily lung tissue) were also used, however due to the spread of bovine spongiform encephalopathy (BSE), bovine-derived heparin was withdrawn from the US market as a precautionary measure [22]. Nevertheless, the reliance on a single species is being increasingly challenged by regulatory agencies worldwide due the following:

- Alternative sources will reduce the dependency on imported porcine material from China. This aspect is particularly relevant due to the multiple problems related to the Chinese pig population over recent last years. These include: (i) The blue ear pig disease (2007) that considerably reduced the pig population across 10 Chinese provinces; (ii) The African swine fever that affected over 100 million pigs (2018–2020) and finally; (iii) In 2008, several heparin batches sourced from China were found to be contaminated with over-sulfated chondroitin sulphate, causing nearly 150 deaths worldwide, prompting the market withdrawal of the affected products [23].
- By increasing the number of alternative heparin sources, any events regarding the adulteration or contamination by viruses and diseases should not have the devastating consequences on the supply chain previously experienced [22, 24].
- The unique reliance on porcine mucosal heparin (PMH) deeply constrains its use in a substantial part of the global population, due to ethical and religious reasons [24].
- Additionally, recent literature has highlighted the sustainability and carbon footprint implications associated with porcine-derived heparin production. It is estimated that 1 kg of porcine intestinal mucosa yields only 160–260 mg of crude heparin, as multiple purification steps are required to isolate heparin from the biological matrix, including enzymatic digestion, filtration, and resin complexation [25]. The production of heparin was estimated to contribute with 52.0 megatonnes of CO_2_ emissions in 2015, which is surprisingly higher value compared to the 46.2 megatonnes of CO2 emissions generated by the automotive industry [25]. As heparin consumption is expected to increase in the coming years, several pharmaceutical industry organisations have pledged their commitment to reducing short-term greenhouse gas emissions. Proposed strategies to achieve this include optimizing the pharmaceutical processes involved in the sourcing, extraction, and purification of crude heparin from porcine intestines through the adoption of more efficient extraction methods, alongside additional measures reviewed in [25].
- Bovine lung is a relatively clean organ, when compared to the intestine. Furthermore, countries like Brazil and Argentina have been commercializing bovine lung heparin (BLH), with a well-established safety record [11, 22], and recently, both India and China have also started the production of BLH [22].
- The authorization of BLH, is expected to increase the value of livestock cattle, from distinct territories [22]. Furthermore, worldwide, there are an estimated 1.5 billion cattle, alongside 1.2 billion sheep. Together, these sources represent a promising alternative to pigs for the supply of heparin [24].

These observations underpin the importance of diversifying the global heparin supply chain. However, to successfully achieve this, interspecies differences need to be taken into account. Given heparin’s structural heterogeneity, the development of good manufacturing process capabilities and related quality controls for the alternative sources is essential, as specified by the FDA in 2025 [26].

As previously highlighted, the most promising alternative heparin source is BLH, which has the following reported differences with PMH:

- BLH is about two-thirds as potent as PMH as an anti-coagulant, as the distribution of the sulphate groups in the polysaccharide chains differs. To overcome this difference, it would be necessary to clinically adjust the dose when used as an anti-coagulant. However, for anti-inflammatory or other effects, the differences between BLH and PMH would require further assessment (as described in the Regulatory Implications section), to determine if dose adjustment is needed.
- The degree of sulphation, and the molecular weight distribution;
- BLH has lower anti-Xa activity than PMH.

For the anticoagulant activity, these differences are not expected to pose an additional risk to patients, given the length of historical use of BLH in the US (60 years), together with the continuing use in South America (Brazil and Argentine) over the last 40 years. Furthermore, studies indicate that if BLH potency concentration is adjusted to PMH potency (e.g. when vialed), identical concentration-response curves in the activated partial thromboplastin time (aPTT) and anti-protease assays are attained [22]. However, for other pharmacological effects, such as anti-inflammatory or antiviral activities, the impact of different heparin sources remains insufficiently explored and requires further investigation [13].

## Current burden of respiratory diseases

The need for the development of new treatments for lung diseases, with an emphasis on prevention, early detection and early treatment has been highlighted by the latest health estimates from the World Health Organisation (WHO) which show that three of the top six worldwide causes of death are lung diseases: Lower respiratory tract infections, lung cancer and COPD [27]. The global burden of lung disease and the importance of a more coordinated respiratory disease research community has been underlined by the recent SARS-CoV-2 pandemic. Recent Office for National Statistics (ONS) data for the UK has revealed that 1.9 million people are suffering from symptoms of long SARS-CoV-2 [28] with SARS-CoV-2 related lung fibrosis potentially increasing the number of patients suffering from interstitial lung disease [29]. Furthermore, the weight of the pandemic has noticeably altered access to clinical pathways delaying early and preventative interventions for patients with respiratory diseases [29].

Pathophysiologically, global burden of lung disease is often a result of the failure of the immune system to defend itself against infection or from disproportionate inflammatory responses against foreign and inappropriate stimuli. It is fair to argue that the most obvious interventions essential to decrease the current burden of lung disease would be reducing exposure to tobacco smoke and air pollutants as a major risk factor of cancer, infections and COPD. Although it can be agreed that changes in environmental exposure will alter disease burden in the long term. However, much remains to be done, especially with regards to the increasing use of vaping products which may have longer-term effects on respiratory health [30, 31]. While there have been major advances in our understanding of the genetics and mechanisms of inflammatory diseases there is unfortunately still a major need to develop novel anti-inflammatory drugs to provide an alternative to corticosteroids. Against this background there is a growing amount of evidence for using inhaled heparin as a treatment for a range of respiratory diseases. Currently, there are a range of heparin products licensed for clinical use or in development for other indications (see Table 1), but none of these are licensed for inhalation for the treatment of respiratory diseases.

**Table 1:** Heparin products licenced for clinical use and in development.

Type of Heparin	Trial stage	Treatment	Structure	Activity	Additional Information
Unfractionated Heparin (UFH)	Clinical	Thrombosis, pulmonary embolism, DVT and ACS	Heterogeneous mixture of GAGs	Inhibits thrombin and factor Xa via antithrombin III	Requires monitoring – risk of Heparin-Induced Thrombocytopenia
Low molecular weight Heparin (LMWH)	Clinical	DVT, PE	Smaller fragments from UFH via depolymerisation	Primarily inhibits factor Xa – minimal effect on thrombin	Reduced risk HIT, e.g. Enoxaparin, Dalteparin, Tinzaparin
Fondaparinux	Clinical	DVT, PE	Synthetic pentasaccharide	Inhibition of factor Xa (selective	Less risk of HIT, minimal interaction with CXCL4. Does not require routine monitoring
Bemiparin	Clinical	DVT, PE	LMWH	Inhibits factor Xa	Only requires once-daily dosing – improved half-life
Nadroparin	Clinical	DVT, PE	LMWH	Inhibits factor Xa	
Pentasaccharide mimetics	Preclinical	Thrombosis, Cancer	Synthetic mimics of the antithrombin – binding site of heparin	Target specific factor Xa inhibition	Under investigation for enhanced efficacy and safety
Sulodexide	Clinical (limited)	Diabetic microangiopathy	GAGs combination	Primarily inhibits factor Xa – reported to have anti-inflammatory properties	
Ultra-low molecular weight Heparin (ULMWH)	Preclinical	Thrombosis and reported potential for cancer treatment	Very small fragments of heparin	Highly specific inhibition of factor Xa	Reducing side effects as well as improving the current bioavailability of heparin
Heparanase inhibitors	Preclinical	Cancer, Inflammatory diseases	Inhibitors that target heparinase enzyme	Inhibit the effects of heparanse (cleave GAGs) – possibility to reduce metastasis	Currently investigated for cancer and anti-inflammatory effects
Heparin analogues/mimetics	Preclinical	Thrombotic disorder, potentially cancer therapy	Modified or synthetic heparins	Target specific factor Xa	Ongoing studies for targeted activity as well as reducing side effects.

### Anti-inflammatory properties of Heparin in the context of inflammatory Diseases of the lung

The wide range of pharmacological effects of Heparin beyond its long recognised anti-inflammatory actions, has been reviewed extensively elsewhere [2, 3]. The beneficial anti-inflammatory effects of heparin were first accredited to inhibition of complement, angiogenesis, adhesion molecules, growth factors and chemokines [32–34].

Heparin’s anti-inflammatory effects are also attributed to its ability to inhibit various inflammatory mediators, such as elastase, histamine and tissue factor pathway inhibitor, and it is now recognised that heparin has the ability to bind to over 400 proinflammatory mediators, cytokines and adhesion molecules, many of which are cationic in nature and/or have heparin binding domains in their tertiary protein/peptide structures [1, 3]. Recent studies also suggest heparin may inhibit the translocation of NF-κB from the cytoplasm to the nucleus, a key step in the activation of inflammatory responses. This modulation can lead to decreased expression of pro-inflammatory genes. In addition to binding a number of adhesion molecules, heparin has been shown to inhibit the ability of adhesion molecules such as P-selectin and L-selectin to recognise their counter ligands essential for the recruitment of leukocytes to sites of inflammation [6]. By binding adhesion molecules, heparin can decrease the infiltration of inflammatory cells which has been demonstrated in a wide range of inflammation models (reviewed in [1, 3]). Interestingly, heparin has also been suggested to upregulate apoptosis of inflammatory cells through pathways involving TNF-α and NF-κB, which can help reduce the number of activated inflammatory cells in tissues [3, 4]. In addition, Heparin also regulates multiple steps in the complement cascade, inhibiting the activity of both the alternative and classical pathways of complement activation [3, 4]. This action reduces the generation of pro-inflammatory complement derived peptides that recruit and activate phagocytes [3]. Heparin can disrupt the formation of NETs, which are networks of extracellular fibres that trap pathogens but can also contribute to tissue damage and inflammation [8]. Heparin can bind to and neutralize neutrophil and eosinophil-derived cationic proteins that contribute to inflammation, further reducing tissue damage [1, 35]. Lastly studies have suggested that heparin may enhance the activity of antiproteases, such as secretory leukocyte protease inhibitor (SLPI) and alpha-1-antitrypsin, which help to protect tissues from oxidative damage associated with inflammation [11].

The effects of heparin are also dependent on its form, for example UFH exhibits multiple anti-inflammatory effects, including inhibition of leukocyte adhesion, migration, and the neutralization of inflammatory mediators [1, 3, 6, 7]. However, its anticoagulant effects can complicate its use in certain clinical scenarios [1]. While LMWH retains some anti-inflammatory properties similar to UFH, its effects may be less pronounced due to its lower molecular weight and altered binding characteristics [3, 35]. It may still inhibit leukocyte recruitment and modulate inflammatory responses, but may not be as effective as UFH in some contexts [35]. However, there are also non-anticoagulant derivatives, such as ODSH (2-O,3-O-desulfated heparin), specifically designed to maximise anti-inflammatory effects while minimising anticoagulation [1]. They can inhibit inflammatory cytokine synthesis, disrupt NETs, and neutralise tissue-damaging mediators without the risk of bleeding associated with anticoagulant activity [8]. Studies have explored the use of novel heparin analogues and heparin-like molecules (glycomimetics) with reduced anticoagulant activity but potent anti-inflammatory effects for the treatment of inflammatory conditions. These novel compounds derived from heparin or that mimic certain actions of heparin offer the potential to target specific inflammatory pathways while minimising the risk of haemorrhage associated with traditional heparin therapy [1, 3].

This review will serve to discuss the anti-inflammatory properties of heparin in different disease states. Although it is appreciated that heparin is known to be of use in treating numerous inflammatory diseases without the need its anticoagulant effects [3, 36], this review will focus on the promise of using inhaled heparin to treat respiratory diseases, specifically, respiratory infections such as SARs-CoV-2, COPD, Asthma, ARDS and cystic fibrosis.

### Respiratory infections

Emerging evidence suggests that heparin may hold therapeutic potential in the management of severe respiratory infections, particularly those caused by viral pathogens such as the influenza virus, respiratory syncytial virus (RSV), and, most notably, SARS-CoV-2 [11, 37]. A key step in viral infection involves the binding of the virus to heparan sulphate (HS) receptors, which, as previously discussed, are on the surface of most mammalian cells. These receptors play diverse physiological roles, including mediating host–pathogen interactions. Structurally, HS closely resembles heparin, differing mainly in a lower degree of sulphation and iduronic acid content [5]. This similarity enables heparin to act as an HS mimetic, competitively inhibiting the interaction between viral proteins and HS on host cell surfaces. Consequently, heparin’s structural resemblance to HS underlies its potential utility in treating infectious diseases caused not only by viruses but also by bacteria and fungi [5]. However, its anti-inflammatory and anticoagulant properties make heparin particularly promising for viral respiratory infections, with numerous studies and clinical trials highlighting its clinical benefits, especially in the context of SARS-CoV-2 infection [5, 13, 38–40].

In addition to the binding to HS receptors, heparin has been shown to bind to the spike protein of SARS-CoV-2, thus blocking its interaction with the ACE2 receptor, which is crucial for the viral entry into epithelial cells [41]. This event reduces the severity of the hyper inflammatory response and the alveolar coagulation associated with SARS-COV-2 infection [42]. Interestingly, heparin plays a role in protecting the endothelial glycocalyx [18]. SARS-CoV-2 can lead to the disruption of the endothelial glycocalyx, a protective layer on the surface of blood vessels that plays a crucial role in maintaining vascular integrity and regulating inflammation [1, 43, 44]. Heparin can help preserve this glycocalyx and we have recently shown that mast cell derived heparin can restore the integrity of the vascular endothelium [16], thereby contributing to a reduction in vascular permeability and inflammation by this drug. This protective effect is particularly important in ARDS, a severe complication of COVID-19 characterized by widespread inflammation and lung injury. In addition, heparin may modulate the levels of pro-inflammatory cytokines and chemokines that are elevated in COVID-19 patients [5, 45]. By binding to these inflammatory mediators, heparin can reduce their activity and help restore a more balanced immune response. This modulation can lead to decreased recruitment of inflammatory cells to the lungs and other tissues, thereby alleviating the severity of the inflammatory response [46].

The ability of SARS-CoV-2 to induce respiratory complications has been well described; however, what has not been widely discussed is the potential of heparin to reduce the complications induced by SARs-CoV-2 infection. The anti-viral, anti-inflammatory and anti-coagulant activities of heparin when administered through nebulization have shown to be safe and effective in improving oxygenation levels, alongside with lower rate of intubation and requirement for invasive mechanical ventilation in hospitalised SARS-CoV-2 infected patients [12, 13]. A retrospective study indicated that inhaled UFH at doses up to 100,000 U daily did not induce clinically relevant systemic anticoagulation and were associated with reduced lung injury [47]. Other studies have shown that inhaled heparin can improve hypoxemia and reduce the need for mechanical ventilation in patients with ARDS related to SARs-CoV-2, suggesting a significant role in managing the inflammatory aspects of the disease [38, 48, 49].

### COPD

Heparin inhibits inflammatory cell recruitment into tissues such as the lung [1, 36, 50, 51]. Heparin’s anti-inflammatory properties also make it a promising option for treating COPD. Its effectiveness in modulating inflammatory responses, decreasing mucus production, and addressing oxidative stress all contribute to its potential as a therapeutic agent for managing COPD [14]. By targeting the inflammatory pathways involved in COPD pathogenesis, including the NF-κB pathway, heparin may help mitigate airway inflammation, reduce exacerbations, and improve lung function in patients with COPD. Furthermore, its anti-inflammatory effects on lung tissue can contribute to the reduction of mucus production and the prevention of airway narrowing, which are characteristic features of COPD [35].

In addition, heparin’s anti-inflammatory properties may help mitigate the systemic inflammation often seen in COPD when administered systemically, by inhibiting inflammatory cytokines and adhesion molecules, potentially leading to improved lung function and reduced exacerbation rates [52]. The use of LMWH has also been investigated, with some studies suggesting it may reduce systemic inflammation and acute lung injury in animal models [53, 54]. Heparin has protective effects on the vascular endothelium, which can be compromised in COPD due to chronic inflammation and oxidative stress. By maintaining endothelial integrity, heparin may improve overall lung function and reduce the risk of cardiovascular complications associated with COPD. Furthermore, one of the significant contributors to lung damage in COPD is the activity of neutrophil elastase, an enzyme that breaks down elastin in the lung tissue [35, 55]. Heparin can inhibit neutrophil elastase activity, potentially reducing proteolytic damage and preserving lung structure. Lastly, heparin may have mucolytic properties, helping to improve mucus clearance in the airways [21, 56]. By inhibiting the expression of mucin genes and reducing the production of mucus, heparin can help improve airway patency and reduce symptoms such as coughing and wheezing. This can be particularly beneficial for COPD patients who often experience mucus hypersecretion and impaired mucus clearance [11, 57].

Clinically, several studies have explored the effects of heparin in patients with COPD. For instance, nebulized heparin increased the number of ventilator-free days in patients with acute exacerbations of COPD, suggesting a potential role in improving respiratory function and reducing the severity of exacerbations [21, 58]. Clinical trials have also indicated that inhaled heparin can improve lung function in patients with moderate to severe COPD. For instance, a study reported significant improvements in forced vital capacity (FVC) and forced expiratory volume in one second (FEV1) after treatment with nebulized unfractionated heparin [59]. A double-blind trial in the 1960s indicated that nebulized heparin could lead to significantly more ventilator-free days in intubated patients with acute exacerbations of COPD. However, results can vary, and some studies have reported no significant benefits, highlighting the need for further research to establish optimal dosing and administration methods [60].

Heparin can be used safely in conjunction with other COPD treatments, such as bronchodilators and corticosteroids, to enhance overall therapeutic effects. For example, studies have shown that co-administering nebulized heparin with salbutamol can lead to improved outcomes compared to salbutamol alone [58] and when administered to patients with COPD on top of standard of care [59]. Given the wealth of clinical data on the effectiveness of inhaled heparin, it is somewhat surprising that there is currently no approved formulation. Therefore, there is ongoing research exploring novel formulations and delivery methods towards developing an approved inhaled formulation of heparin.

### Asthma

Asthma is a common chronic airway disorder. However, its presentation is complex and characterised by a multitude of recurring features: underlying inflammation, mucous hypersecretion, airflow obstruction and bronchial hyper responsiveness [14, 61]. Asthma is characterized by acute and chronic airway inflammation, which leads to symptoms such as wheezing, shortness of breath, and coughing.

Heparin has been shown to help patients with asthma [5]. The initial studies some 60 years ago described improvement in asthma symptoms as a result of intravenous heparin [62, 63]. These studies showed an elimination of bronchospasm and obstructing secretions although they were unable to confirm the mechanism(s) responsible. In addition, inhaled heparin has been shown to reduce allergen induced bronchospasm in allergic asthmatics [64, 65] and exercise-induced bronchospasm [66]. Several studies have suggested that heparin can inhibit the release of pro-inflammatory mediators and cytokines involved in the activation and recruitment of inflammatory cells in the airways such as IL-5 that may contribute to its beneficial effects in patients with asthma [2, 3]. Bendstrup and Jensen reported two individual cases; Firstly, a 57-year-old female who presented with progressive allergic asthma for 6 years and a 67-year-old female who had suffered from allergic asthma since childhood. Both patients were corticosteroid-resistant but positively responded to inhaled heparin during exacerbations of their asthma [67].

In asthma, persistent inflammation can lead to airway remodelling, characterized by structural changes in the airway walls and subepithelial fibrosis. Heparin’s anti-inflammatory properties may confer protection against airway remodelling in asthma by modulating the production of extracellular matrix components and growth factors, thereby contributing to the preservation of lung function [14, 68]. Airway hyper responsiveness is a hallmark of asthma and is closely linked to the underlying inflammation. Heparin’s anti-inflammatory properties may play a role in regulating airway hyper responsiveness by targeting the inflammatory pathways that contribute to this heightened airway reactivity [14]. Through its modulation of inflammatory mediators, heparin has the potential to mitigate airway hyper responsiveness in asthma [21]. Furthermore, similar to its effects in patients with COPD, heparin has been shown to modulate the activity of inflammatory cells in asthma [69]. By regulating the activation and recruitment of neutrophils, eosinophils, and mast cells, heparin may help mitigate the chronic inflammatory response in the airways, contributing to the attenuation of asthma symptoms and exacerbations. Heparin’s ability to inhibit mast cell degranulation and reduce the release of histamine and other mediators, may help alleviate asthma symptoms and improve airway function [9]. This effect has been suggested to be via heparin inhibiting the intracellular signalling molecule, IP3 [9]. The use of heparin in combination with other asthma therapies, could potentially enhance treatment outcomes, particularly in patients with severe or refractory asthma.

The variability in individual responses to heparin and the need for further research to establish optimal dosing and administration routes are important considerations. Ongoing research is focused on optimizing the formulation and delivery of heparin for inhalation, as well as exploring its long-term effects and potential in combination therapies for asthma management. There is also interest in developing non-anticoagulant derivatives of heparin that retain its anti-inflammatory properties for asthma treatment.

### ARDS and Acute Lung Injury

ARDS and lung injury are characterised by pulmonary activated coagulation and can develop in patients of all ages [70]. ARDS originates from damage, either directly or indirectly, to the lungs as a consequence of a range of situations such as trauma, sepsis or pneumonia. The lungs are characterised by increased procoagulant factors, increasing protein permeability and inflammation [71]. The pathophysiology of the lungs results in the activation of pulmonary macrophages towards a pro-inflammatory phenotype, increasing intravascular and extravascular platelets, neutrophils and fibrin highlighting the need for novel treatments having a broad range of pharmacological actions such as heparin [72, 73].

In the context of ARDS, heparin’s anti-inflammatory properties hold significant potential for attenuating the widespread inflammation in the lungs. By inhibiting the release of pro-inflammatory mediators and cytokines, heparin may aid in reducing the inflammatory cascade that contributes to lung damage and impaired gas exchange in ARDS [13, 47]. Heparin may exert its effects in ARDS through several mechanisms, including the inhibition of neutrophil elastase, modulation of inflammatory cell recruitment, and disruption of neutrophil extracellular traps (NETs), which are implicated in lung injury [7, 8]. Moreover, heparin’s ability to modulate the activity of inflammatory cells and inhibit the release of pro-inflammatory cytokines further supports its potential role in managing the hyperinflammatory state and mitigating the inflammatory response characteristic of ARDS. Furthermore, one of the hallmark features of ARDS is the disruption of the endothelial barrier in the lungs, leading to increased permeability and pulmonary oedema [74]. Heparin’s capacity to preserve endothelial barrier function and prevent vascular leakage may offer protective effects against the development and progression of pulmonary oedema in ARDS [75–77]. By maintaining the integrity of the endothelial barrier, heparin could help mitigate the severity of lung injury and respiratory compromise in ARDS patients [37, 78].

In addition to its anti-inflammatory effects, heparin’s anticoagulant and fibrinolytic properties are of particular interest in the context of ARDS [77]. The dysregulation of coagulation and fibrinolysis pathways is intricately linked to the pathophysiology of ARDS, and heparin’s ability to modulate these processes may contribute to the prevention of microvascular thrombosis and fibrin deposition within the lungs. By mitigating coagulation-related complications, heparin could potentially alleviate the severity of ARDS and improve outcomes for affected individuals [1, 76].

Clinically, a landmark multicentre clinical study (CHARLI) reported that nebulized heparin is well tolerated in patients with ARDS or those at risk of developing it [47]. While nebulized heparin did not significantly improve self-reported performance and daily physical activities at day 60, it did show a significant impact on exploratory endpoints, such as reducing the number of patients developing ARDS and improving recovery rates [47]. The study indicated that nebulized heparin, when administered alongside standard care, resulted in less deterioration in Acute Lung Injury scores and allowed more patients to return home by day 60 compared to those receiving a placebo [47]. The local delivery of heparin via nebulization may provide targeted effects in the lungs, potentially leading to improved outcomes in ARDS patients without significant systemic anticoagulation effects [13, 47]. Heparin’s ability to reduce inflammation and improve lung function could be particularly beneficial in the context of ARDS, where inflammation plays a critical role in disease progression.

Regarding Acute Lung Injury and smoke inhalation, there are a plethora of both nonclinical and clinical evidence supporting therapeutic benefits of UFH, owing to its anti-inflammatory, mucoactive, and antimicrobial effects. In nonclinical models of acute lung injury and systemic inflammation established across several species such as rats, mice and sheep, UFH has consistently been able to modulate inflammation, infection, mucus properties, pulmonary coagulation, as well as haemodynamics [79–85]. Clinically, UFH has been associated with improvements in surrogate respiratory outcomes, such as lung injury scores, airway resistance, compliance, hypoxia scores, oxygenation, and an increase in ventilator-free and ICU-free days, leading to earlier liberation from mechanical ventilation. A survival benefit was observed in specific subgroups, particularly in patients with higher disease severity (e.g., APACHE III score > 35), and when UFH was combined with adjunctive therapies such as acetylcysteine and albuterol [86–90].

However, there are challenges to consider. While the findings from studies are promising, the optimal dosing regimen, administration routes, and long-term effects of heparin in ARDS treatment are not fully understood. The timing of heparin administration is likely critical. Similar to other therapies, such as corticosteroids, early delivery, particularly before severe lung injury develops or intubation becomes necessary, may be key to preventing disease progression [91]. Once ARDS progresses to a late fibrotic stage, the therapeutic window is likely reduced. However further clinical evidence is needed to establish the optimal timing of heparin administration.

Further research is needed to establish the efficacy and safety of nebulized heparin in larger, controlled trials to confirm its benefits in ARDS patients.

### Cystic fibrosis

CF is a genetic disorder characterized by the production of thick, sticky mucus that can obstruct the airways and lead to chronic respiratory infections and inflammation. While heparin is primarily recognized for its role in anticoagulation, its potential therapeutic effects in CF extend beyond its anticoagulant properties [1]. By targeting specific inflammatory mediators and cellular responses involved in the disease pathogenesis, heparin has the potential to attenuate the exaggerated inflammatory response in the airways of individuals with cystic fibrosis [10]. This modulation of inflammation could help alleviate airway obstruction and reduce the frequency of respiratory infections in affected individuals.

Several studies have highlighted the potential benefits of using heparin to treat CF as it may lead to reduced bacterial colonisation [92, 93]. Heparin has an indirect antimicrobial effect, particularly against bacterial pathogens commonly associated with CF, such as *Pseudomonas aeruginosa* [94]. It can inhibit the binding of respiratory viruses to epithelial cells, which may help reduce the risk of viral infections that can exacerbate CF symptoms [95]. Alleviating the risk of bacterial colonisation in turn could help to decrease the rate of inflammation, which are both critical factors in managing CF. It may also help reduce the exposure of these patients to the fluoroquinolone class of antibiotic, which has a number of side effects, some of which can be severe.

Several clinical studies have investigated the effects of inhaled heparin in CF patients [92, 93]. For instance, inhaled UFH has been shown to reduce sputum neutrophil elastase activity and inflammatory cytokines, indicating its potential to modulate inflammation in the CF airways [57]. This makes it a potentially safe option for CF patients, who are often at risk for bleeding complications due to their underlying condition and treatments [1]. However, results have been mixed, with some studies showing no significant changes in lung function despite improvements in inflammatory markers [1, 3]. Although inhaled heparins were deemed safe, it is fair to note that the variability in the clinical trials indicates the need for further dose ranging and placebo control studies as well as alternative formations. Inhaled heparin formulations, such as those co-sprayed with L-leucine, have been developed to enhance delivery and efficacy in the lungs, potentially providing a more effective treatment option for CF patients [96, 97]. In addition to new formulations, another promising avenue might be the use of heparin derivatives with low anticoagulant activity, such as N-acetyl heparin and glycol-split heparin [94]. These derivatives have shown promise in reducing bacterial burden and inflammation in preclinical models of CF.

Studies have suggested that heparin may modulate inflammatory pathways and mucus production in the context of cystic fibrosis. In cystic fibrosis, the excessive production of thick and sticky mucus in the airways leads to recurrent respiratory infections and chronic inflammation [98, 99]. Heparin can act as a mucolytic agent, helping to break down DNA and other components within the thick mucus that is synonymous with CF [11]. By influencing mucus viscosity and clearance, heparin may contribute to reducing airway obstruction and improving respiratory function in individuals with CF [92]. Targeting mucus production and clearance represents a promising avenue for therapeutic intervention in the management of CF.

Exploring the use of heparin in combination with other mucolytic agents or anti-inflammatory therapies could lead to synergistic effects, enhancing its therapeutic impact in cystic fibrosis. By targeting multiple aspects of the disease pathology, such combination approaches may result in improved clinical outcomes and respiratory function for individuals with CF [11]. Continued exploration of heparin’s potential in cystic fibrosis management, particularly in the context of modulating inflammatory processes and mucus production, holds promise for advancing the development of novel therapeutic strategies tailored to the unique challenges posed by this complex genetic respiratory condition. This includes further investigations into optimizing heparin dosing regimens, evaluating its long-term safety profile, and assessing its potential synergistic effects with other therapeutic interventions in CF. Furthermore, investigating the potential use of heparin in combination with other anti-inflammatory agents or emerging therapies targeting specific disease mechanisms could lead to more comprehensive and individualized treatment approaches for the treatment of respiratory diseases.

## Safety of inhaled heparin

Since the COVID-19 pandemic, there has been a renewed interest in nebulized heparin, with an expanding body of clinical studies supporting its efficacy and safety profile [13, 14, 49, 78, 100]. This builds on earlier evidence in acute lung injury and ARDS, where nebulized UFH has consistently shown no increase in systemic bleeding risk, despite occasional, clinically insignificant increases in aPTT [47, 101, 102]. Similar findings have been also reported in smoke inhalation and burn-related lung injury, with no increase in bleeding events or relevant coagulation abnormalities [89, 90, 103, 104]. In chronic respiratory diseases, such as asthma, cystic fibrosis, idiopathic pulmonary fibrosis, and COPD, the safety profile of inhaled heparin is similarly reassuring, with no clinically meaningful effects on coagulation, bleeding, or thrombocytopenia, even at high doses (up to 150,000 IU/day) [21, 56].

In COVID-19 populations, including both intubated and non-intubated patients, studies consistently report no pulmonary or systemic bleeding, with only minor, clinically insignificant changes in aPPT [13, 49, 78, 100]. The INHALE-HEP meta-trial, evaluating doses up to 25,000 IU every 6 h, further confirmed the absence of bleeding concerns, even when nebulized heparin was administered alongside with systemic anticoagulation drugs [13].

In summary, inhaled heparin has demonstrated a consistently favourable safety profile across a wide range of respiratory diseases, including at doses up to 400,000 IU/day [102], as supported by subsequent studies [1, 11, 13, 14, 47, 78].

## Pulmonary delivery of Heparin

Local administration of heparin by inhalation offers a strategy to target respiratory diseases directly within the lungs, maximising lung-specific effects while limiting systemic anticoagulation and bleeding risk [69]. In particular, being a very anionic polymer, bioavailability of heparin is very poor which helps improve the safety margin when it is administered by this route. It is also important to consider that it is not just the formulation of heparin that is important, but also the correct choice of pulmonary drug delivery devices—such as nebulisers, dry powder inhalers, metered-dose inhalers and soft-mist inhalers. These devices, which ensure maximum clinical benefit, also offer the advantage of enabling patient self-administration, thereby improving adherence to treatment. Furthermore, they represent a viable strategy for patients requiring oxygen therapy or respiratory support, including high-flow nasal oxygen (HFNO), as well as non-invasive and invasive mechanical ventilation [69, 105].

### Physicochemical and Biological Challenges of the Pulmonary Delivery of Heparin

Despite its therapeutic promise, optimising the pulmonary delivery of heparin is challenging due to several of its intrinsic physiochemical properties:

1. High molecular weight
  UFH is a heterogeneous polysaccharide mixture (≈ 5,000–30,000 Da), limiting tissue penetration. Low-molecular-weight heparins partially mitigate this limitation but remain highly charged and hydrophilic.
2. High negative charge (polyanionic nature)
  Due to its highly sulphated composition, heparin is one of the most negatively charged molecules in biology and consequently, is able to bind to a wide range of positively charged biological materials [19]. However, from a formulation perspective, this same feature results in strong electrostatic repulsion from negatively charged cell membranes, binding to cationic proteins in mucus, and adsorption to positively charged nebuliser components, ultimately leading to drug loss during aerosolisation.
3. Extreme hydrophilicity
  Poor lipid membrane permeability limits passive diffusion, increasing reliance on inefficient paracellular or endocytotic transport and thus susceptibility to rapid mucociliary clearance.
4. Viscosity and solution behaviour
  At high concentrations, heparin solutions become viscous and adhesive, impairing nebuliser performance, increasing aerosol droplet size, and reducing deep-lung deposition.
5. Stability concerns
  Heparin may degrade during ultrasonic nebulisation, heat exposure, or prolonged storage, necessitating careful formulation and device compatibility.
6. Mucus interactions
  Binding to mucins and airway proteins may trap heparin in mucus, reducing epithelial exposure and shortening lung residence time.

Furthermore, several defence mechanisms in the airways may pose significant challenges to an efficient drug delivery following pulmonary administration. From a mechanical point of view, the impact of particles/droplets in the mouth, nose, large airways, will compromise drug delivery to peripheral lung regions. Moreover, in respiratory diseases, airway narrowing, mucus hypersecretion, and mucus plugging create additional mechanical barriers that must be considered [106]. Lung mucociliary clearance should also be accounted for, as it may be responsible for moving the drug away from target sites. In addition to these mechanical barriers, the airways present chemical and immunological hurdles to drug absorption into tissues, which may be particularly challenging for heparin delivery. These include the presence of proteolytic enzymes and surfactants that can hydrolyse peptides and proteins in the lungs, resulting in their inactivation. Furthermore, undissolved drug particles may be engulfed by alveolar macrophages, resulting in their removal from the lungs [106, 107].

These factors collectively complicate efficient lung delivery and motivate exploration of improved formulations and delivery systems that allow for an improved flow rate, more efficient nebulisation and better delivery of the drug throughout the respiratory tract.

### Pulmonary delivery modalities

There are four main types of delivery systems for respiratory dosage forms that are suitable for pulmonary delivery of heparin: (1) dry powder inhalers (DPIs), (2) metered-dose inhalers (MDIs), (3) soft mist inhalers (SMIs) and (4) nebulisers [105, 108]. A brief overview of each device type and their use with heparin-containing formulations is discussed below.

#### Dry powder inhalers (DPI)

DPIs administer drug as a pure powder, or as a mixture with inert carriers, such as lactose, glucose or mannitol [109]. The powder flow is triggered whenever the patient inhales, not requiring any propellant [105, 108]. By relying on dry powder formulations, DPIs can offer an increased chemical stability when compared to their liquid-based counterparts. For protein and peptide drugs delivery, like heparin, this feature is appealing due to higher susceptibility to aqueous degradation overtime [109, 110].

As the formulation is simple, this dosage form does not require cold chain storage or powder reassembly; furthermore, no bulky compressor or compressed air source is required, in contrast to nebulisers, which will be detailed in the following sections. This dosage form demonstrates favourable flow and dispersion profiles, deep lung deposition, along with improved patient compliance when compared to nebulisers [111]. All these features make DPIs attractive dosage forms for heparin delivery, and unsurprisingly there are several reports investigating the delivery of heparin through DPIs (see Table 2).

**Table 2:** Delivery of heparin via DPIs.

DPIs Formulation strategy	Indication	Strength	Main outcomes	Reference
Heparin-azithromycin microparticles	Bacterial lung infections associated with COVID-19.	Heparin sodium: 75% to 95% AZM: 5% Leucine 0 to 20% (w/w)	High antiviral activity Comparable or superior antibacterial efficacy to unprocessed azithromycin Favorable safety profile up to 50 µg/ mL Optimized lung deposition	[111]
Lactose-based dry powder formulation of Enoxaparin	Anticoagulation	0.5 U/ mg	Formulation was 1.5-fold as effective, when compared to inhaled enoxaparin solution Smaller particle size yielded higher pulmonary absorption	[109].
Spray-dried spherical micronized UFH particles	Cystic Fibrosis	Pure UFH	Production of respirable UFH particles suitable for pulmonary delivery, particle size 1–5 μm and spherical morphology	[97]
Spray-dried heparin sodium co-formulated with chitosan and L-leucine	Respiratory inflammatory and infectious diseases	0.2% (w/v)	The combination of chitosan and L-leucine improved formulation performance compared to single-excipient systems - aerosolization performance, storage stability, and sustained drug release.	[112]
Co-jet-milling heparin sodium co-formulated with magnesium stearate	Pulmonary infectious diseases and complications (pulmonary thromboembolism)	180 IU/mg	Co-milling with magnesium stearate improved the emitted rate and emitted fine particle fraction, alongside with formulation stability This approach enables the manufacture of high dose carrier-free heparin DPI formulations	[113]
LMWH PE-PLGA microparticles	Anticoagulation	100 mg/ml	High LMWH entrapment efficiency using sodium chloride as an osmotic agent in external aqueous phase; Particle size and aerodynamic diameter suitable for inhalation; Reduced uptake by alveolar macrophages, enhanced lung deposition, sustained drug release, prolonged pharmacokinetic and pharmacodynamic effects after intratracheal administration (rats)	[114]
LMWH Porous microspheres	Anticoagulation	61 U/mg	Improved entrapment efficiency; Particle size, aerodynamic diameter and biocompatibility suitable for inhalation; Sustained LMWH release in vitro and in vivo for up to 24 h with prolonged anti-factor Xa activity versus plain inhaled LMWH.	[115]

Although not all of the reported formulations in Table 2 are specifically intended for the treatment of respiratory diseases, they all focus on the pulmonary delivery of heparin, or its derivatives, via DPI dosage forms. The formulation strategies range from simple carrier-based systems to advanced spray-dried, co-milled, or polymeric microparticles. Across these studies, key performance parameters include particle size compatible with this administration route, high aerosolization efficiency, and enhanced lung deposition. Collectively, these findings highlight the versatility of DPI formulations in enabling an effective pulmonary delivery of heparin-based therapeutics for diverse pulmonary and systemic therapeutic indications. However, these devices also present some disadvantages from a user perspective. DPIs require a strong inspiratory effort and are less effective in patients with severe airflow limitation (e.g. critically ill, patients suffering from severe respiratory illnesses, children and cognitively ill patients). Therefore, for these specific clinical indications, these systems would not be the first choice. Furthermore, from a technological point of view these devices are sensitive to humidity and the sheer stress may denature proteins and complex large molecules, like heparin [116].

#### Pressurized Metered-Dose Inhalers (pMDI)

The pDMI has two distinct components - the propellant and the formulation, in which the API is commonly included as a solution or suspension [108]. These devices comprise three parts: a metal container, filled with formulation, a metering valve, responsible for ensuring a constant spray dose and finally, a nozzle (or mouthpiece) that allows the drug aerosol to be inhaled into the respiratory tract [105]. These dosage forms are portable and display a hand-held design which reinforces patient compliance. Furthermore, pMDI deliver accurate doses with each actuation. However, similarly to DPI, pMDIs require hand–inhalation coordination, making them less convenient for critically ill patients, those with severe respiratory illnesses, paediatric populations, and patients with cognitive impairment. Also, pDMI can lead to excessive drug deposition in the oropharyngeal region, thereby requiring higher dosage levels to achieve a therapeutic effect [116]. The use of pMDIs to deliver heparin may be challenging due to the lack of heparin solubility in non-polar hydrofluoroalkane propellants which are commonly used in these dosage forms.

Hand–inhalation coordination is a common limitation of pMDI. However, the use of spacers can mitigate this issue by improving drug deposition and reducing the need for a strong inspiratory effort or precise hand–mouth coordination [116]. Spacers are add-on devices consisting of a reservoir positioned between the inhaler mouthpiece and the patient’s mouth. Alternatively, valved holding chambers (VHCs) may be used. These incorporate a one-way valve that allows airflow into, but not out of, the patient’s mouth [117]. The use of spacers or VHCs in these delivery systems has demonstrated efficacy comparable to that of nebulisers [118]. Additionally, several advantages have been reported, including lower cost, an improved side-effect profile in paediatric populations, and the easier facilitation of patient and caregiver training in DPI/pMDI use, compared with nebulisers [118].

#### Soft-mist inhalers

Innovations in inhaler technology have resulted in the development of low-velocity spray devices, called soft mist inhalers (SMIs). These are propellant-free inhalers that use mechanical power to deliver single or multiple doses of aerosols as a slow mist. Upon device actuation in SMI, a spring mechanism is triggered, supplying the mechanical energy necessary to propel the formulation through a fine nozzle. During this process, a metered dose of the solution is atomized and split into two fine liquid jets, which collide at a predefined angle. In addition, the aerosol cloud has a prolonged generation time, around 1.5 s [119]. Together, these mechanisms account for two of the main characteristics of DMI: a high fraction of fine particles (65–80%) and a slow-moving aerosol plume through the respiratory tract during deep inhalation [119]. All together, these events are responsible for two of the main features of DMI – a high percentage of fine particles (65–80%) and a slow moving pattern of the solution, through the respiratory pathways, as the patient deep inhales [119]. The reduced velocity of the aerosol mist significantly eases the coordination of actuation and inhalation of SMIs, especially when compared to DPIs [120, 121]. The aforementioned features minimize the so called “ballistic effect” [120, 122]. This means that by decreasing the initial velocity and momentum of the mist, the oropharyngeal impact is significantly reduced [119]. By reducing so, the delivered dose is increased up to more than 50% when compared to DPI, independently of the patient’s inspiratory flow [119].

The formulation in SMIs is in the liquid state and it is stored in an enclosed system, such as a pre-filled syringe or cartridge. These devices are highly portable and compact. When compared to most pMDIs and dry powder inhalers (DPIs), SMIs display a higher fraction of fine particles and, as previously detailed, a distinct aerosol spray pattern - slower and longer than that from pMDIs and DPIs. These features minimize the impact of errors related to hand-lung coordination. Moreover, some SMIs can be used to aerosolize lipid nanoparticle formulations (e.g. rhDNAse, mRNAs) which are not available as other inhalation dosage forms or are sensitive to current aerosolisation methods [120].

A key challenge in developing formulations for SMIs is the effect of fundamental parameters, such as solubility, viscosity, surface tension, density, drug and ion concentrations on aerosol performance [120]. With respect to pulmonary delivery of heparin using soft mist inhalers (SMIs), a key limitation is their relatively small fill volume (approximately 15 µL–1 mL). However, this same characteristic confers high portability, which in turn enhances convenience and supports improved patient compliance [123]. This disadvantage could be surpassed by increasing the heparin dose in the liquid formulation, however the impact on viscosity would need to be carefully monitored [120].

Examples of commercially available or under clinical evaluation SMIs include: Respimat^®^ (Boehringer Ingelheim), Softhale (Softhale N.V), MRX00 (Merxin Ltd), PFSITM (Resyca BV), Aqueous Droplet Inhaler^®^ (ADI) (Pharmaero), Trachospray (Medspray), Ecomyst90^®^ (Aero Pump GmbH), SoftBreezer^®^ (Ursatec GmbH), Pulmospray™ (Resyca BV), among others [120]. While most of these SMIs are designed for the delivery of bronchodilators, there are also examples being used to deliver anaesthetics, nicotine, antibiotics and notably, the Pulmospray™ (Resyca BV) which has also been evaluated for LMWH delivery [124]. In the context of COVID-19 – Induced ARDS, inhaled LMWH was assessed in a phase IIb, open-label clinical trial using this inhaler. Patients with more severe hypoxemia received 4000 IU twice daily for 10 days, while a control group received standard therapy. Treatment resulted in significant improvements in oxygenation and breathing capacity, particularly in patients with severe hypoxemia [125].

#### Nebulisers

Nebulisers form small aerosol droplets that carry the drugs into the lungs, thereby enabling the topical treatment of respiratory diseases, with reduced systemic side effects [126]. Nebulisers, in contrast with pressurized metered-dose inhalers (pMDIs) and dry powder inhalers (DPIs), do not require coordination between inhalation and actuation. This feature makes them useful for pediatric, elderly and non-conscious patients. In fact, nebulization remains the most clinically advanced and extensively studied delivery method, as these devices are widely used in hospitals in ventilated patients. In these circumstances, the nebuliser head is incorporated into the ventilator to administer aerosol therapy [126].

Despite their advantages, nebulisers also present some limitations, such as variable lung deposition, upper airway loss, and frequent dosing requirements. Furthermore, improper cleaning can lead to contamination, posing a significant risk of cross-infection in hospital settings. Regular maintenance requirements may also increase treatment costs. In addition, nebulisers typically require several minutes to deliver an adequate drug dose to the lungs; this delay, together with their bulky design, may reduce patient compliance compared with other respiratory delivery methods [69].

Based on their operating principles, nebulisers can be classified into three types: jet, ultrasonic, and mesh nebulizer [127], which are briefly addressed.

#### Jet nebulisers

The basic functioning principle of jet nebulization is that a compressed gas (i.e. air) is forced through a tubing system, which is in turn connected to a nozzle. The increase in air velocity, alongside with the decrease in the tubing cross-sectional area, create a zone of low pressure around the nozzle (Venturi effect). Further, as the high-velocity jet passes tangentially or coaxially through the nozzle, the pressure drop causes the liquid formulation to rise up on a feed tube from the liquid reservoir. A primary droplet is then formed as an aerosol. Afterwards, larger droplets will collide with the internal components of the device and will return to the reservoir, whilst smaller droplets remain suspended and are emitted as a respirable aerosol [128].

Jet nebulisers are considered the gold standard approach used in the clinic [127]. In fact, current product information on dornase alfa for aerosol therapy in cystic fibrosis patients only recommends six jet nebulisers and one vibrating mesh nebulizer [127].

There are many different types and brands of jet nebulisers available on the market. A few examples include: AeroEclipse II, Assister KN-180, Genki, Hudson T Up-draft, Marquest Acorn II, Medel Clenny, Medel SkyNeb, Mefar 2000, Micromist, Millicon S, Nesco, Nissho, Omron NE, Pari LC Jet Plus, Pari LC Sprint, Pari LC Star, Pari LL, Sidestream, Sidestream Plus, Ventstream, Circulaire, among others. These can be classified into four different categories of Jet nebulisers, those with a corrugated tube, Jet nebulisers with a collection bag, Breath-enhanced jet nebulisers and Breath-actuated jet nebulisers.

For heparin delivery using jet nebulisers, the viscosity of the formulation needs to be carefully balanced. As previously described, jet nebulisers have been used for heparin pulmonary delivery in several clinical studies [13, 56, 100], however, all employed saline-diluted commercial systemic formulations “off label”.

#### Vibrating Mesh

In vibrating mesh nebulisers, the drug formulation passes through a tiny mesh that is responsible for the formation of a fine mist. In essence, these devices can be classified as micropump systems, because the aerosol is formed due to the energy of a vibrating piezo-electric orifice plate, forcing liquid to flow through small apertures of a plate or membrane.

Vibrating mesh nebulisers are portable, patient friendly, display zero residual volume, enable an efficient, rapid and reproducible delivery of small drug volumes to the lungs [128]. Due to these characteristics these devices have sparked interest for pulmonary delivery of expensive protein-based biopharmaceuticals. Furthermore, these nebulisers can be used during any type of respiratory support including mechanical ventilation, high frequency oscillatory ventilation (HFOV), non-invasive ventilation (NIV), continuous positive airway pressure (CPAP) and High Flow Nasal Cannula (HFNC).

Mesh nebulisers can be divided into two types: static (passive) and vibrating (active) nebulisers. In general, active vibrating-mesh nebulisers are more efficient for low viscosity formulations, when compared to jet nebulisers. On the other hand, passive vibrating-mesh devices present comparable performance to jet nebulisers, but are however more efficient when delivering protein solutions. Similarly, to jet nebulisers, vibrating mesh nebulisers have also been used in several clinical trials with nebulized UFH [13, 40, 47, 49, 88].

#### Ultrasonic nebulisers

In ultrasonic nebulisers, acoustic waves are generated by a piezoelectric transducer that converts an electrical signal into an oscillatory mechanical motion. The resultant effect is bulk cavitation and surface interference waves giving rise to droplet formation. Ultrasonic nebulisers promote an increase in solution temperatures to as much as 10 °C above the starting temperature after a 5- to 10-min aerosolization period [128]. Two different classes can be considered: large-volume ultrasonic nebulisers and (2) small-volume ultrasonic nebulisers. The first type is commonly employed to deliver hypertonic saline for sputum induction, whilst small-volume ultrasonic nebulisers are used for delivery of inhaled medications. Ultrasonic nebulisers have many limitations compared to jet nebulisers: more expensive nebulisers display large residual volumes, an inability to aerosolise viscous solutions (and are thus inappropriate for heparin nebulisation) and degradation of heat-sensitive materials (e.g. suspensions and proteins) [127–129].

For ventilated or intubated patients (e.g., resulting from ARDS, smoke inhalation), nebulised heparin offers a feasible delivery route via ventilator circuits — an important advantage over DPIs which depend on patient inspiratory effort. For ambulatory patients (e.g. viral pneumonia, early COVID-19, chronic lung disease), nebulisers may be less convenient and less portable than inhalers; however, the infrastructure, cost, and patient adherence may favor nebulisation in hospital settings. In resource-limited settings (e.g., overwhelmed hospitals), nebulised UFH — given its low cost and broad availability — could be an attractive adjunct therapy, particularly where advanced therapies (e.g., extracorporeal membrane oxygenation, ECMO) are unavailable [89, 130, 131].

A summary of available nebulisers is presented in Table 3.

**Table 3:** Summary of different types of nebulisers. Adapted from [129, 132].

Nebulizer Class	Subclasses	Advantages	Disadvantages	Suitability for UFH delivery
Jet	Jet nebulisers with corrugated tubing Jet nebulisers with a collection bag Breath-enhanced jet nebulisers Breath-actuated jet nebulisers	Easy to use uitable for all ages → Do not require hand–breath coordination Droplet size independent of patient Enable the delivery of high doses	Significant drug losses Difficult to clean → can lead to contamination Limited portability and loud noise Requires a source of compressed air/gas Variability between nebulisers Device preparation required High administration time Large interdevice variability	Have been used in the clinic
Vibrating mesh	Static Vibrating	Fast, portable and quiet Self-contained power source Able to produce finer particles → deliver more drug to the distal airways	Not compatible with viscous liquids or those that crystallize on drying Difficult to clean	Have been used in the clinic
Ultrasonic	Large-volume Small-volume	No coordination required Less noisy Higher output Easy to clean	Not feasible to deliver suspensions Larger particles emitted Possible drug denaturation due to generated heat during nebulization inability to aerosolize viscous solutions	Not ideal —may denature heparin and may cause device blockage

#### Nanoparticle and liposomal systems

Encapsulation of heparin in advanced delivery systems such as liposomes or nano- and microparticles has been proposed to enhance pulmonary delivery by protecting the drug from enzymatic degradation, prolonging lung retention, and enabling sustained local effects [11, 15, 133]. These systems may also improve passive lung targeting and reduce systemic absorption. Table 4 depicts some of the formulation strategies referred in the literature.

**Table 4:** Heparin nanoparticles for pulmonary delivery

Formulation strategy	Indication	Main outcomes	Reference
Chitosan and glycol-Chitosan nanoparticles containing Lipoid S100 and loaded with LMWH, (ionotropic gelation method)	Pulmonary embolism and thromboembolic disorders	Nanoparticles showed high encapsulation efficiency, mucoadhesive properties, and suitable aerosolization for lung delivery	[134]
Mesoporous iron(III) carboxylate MIL-100(Fe) nanoparticles associated with favipiravir and heparin	Anti-viral - AntiSARS-CoV-2 Therapy	Heparin contributed to antiviral activity by binding to the SARS-CoV-2 spike protein and limiting viral entry Displayed local anti-inflammatory and endothelial protective effects, Provided anticoagulant action to counteract pulmonary microthrombosis	[135]
Chitosan–hyaluronic acid nanoparticles loaded with heparin	Asthma	Heparin-loaded CS–HA nanoparticles showed suitable size and positive surface charge for pulmonary delivery, High encapsulation efficiency, and stability; Nanoparticles were efficiently internalized by mast cells and inhibited histamine release ex vivo,	[136]

Despite these examples, the clinical approval of nanoparticles remains challenging due to safety concerns, aerosol and formulation stability, alongside limited *in vitro* and *in vivo* data. In addition, regulatory barriers persist, as key carrier materials such as poly-L-lactide-glycolic acid, poly(lactic acid), and chitosan have not yet been approved by regulators such as the FDA for pulmonary delivery, underscoring the need for further research to balance dosing, inhalation performance, and patient or ventilator compatibility [11, 133].

#### Pharmacokinetics of inhaled heparin

The pharmacokinetics of nebulised heparin in humans is characterized by significant local pulmonary retention with minimal systemic absorption, therefore heparin´s repurposing strategy is a viable one for the treatment of respiratory diseases without inducing a state of systemic anticoagulation, even in patients receiving systemic anti-coagulant therapy.

The high molecular weight alongside the strong negative charge of UFH pose significant challenges for UFH mucosal barrier absorption, especially at nominal doses lower than 8 mg/kg [137]. Studies indicate that inhaled heparin distributes uniformly throughout the lungs [138, 139]. Furthermore, heparin exhibits a prolonged effect, with an estimated alveolar half-life of 28 h, and studies suggest that approximately 40% of the dose remains in the lungs after 24 h. Systemic anticoagulant markers (aPTT, anti-Xa) may rise at high inhaled doses but are generally modest and dose-dependent [11, 131].

While the published literature focuses heavily on pulmonary kinetics, the general metabolism of heparin in the circulation follows these pathways:

- UFH Clearance Mechanism: UFH is cleared through a saturable, non-renal route involving endocytosis by scavenger receptors (specifically the HARE receptor) on endothelial cells in the liver and lymph nodes [11].
- Intracellular Metabolism: Once internalised by cells, heparin undergoes lysosomal breakdown through a series of specific enzymes, including various sulfatases and hydrolases that dismantle its molecular structure [1].
- Clinical studies indicate that doses up to 100,000 IU daily do not induce clinically relevant changes in systemic coagulation parameters, such as the aPTT [47, 131].

## Regulatory implications

From a regulatory perspective, heparin products may be classified differently depending on the regulatory authority. For instance, the US-FDA considers UFH a drug, and not a biologic, and hence requires applicants to follow the NDA pathway as specified under Sect. 505(b)(1) and 505(b)(2) of FDCA, and not the biologics licensing pathway [140]. Conversely, the EMA, WHO and several other regulatory agencies consider UFH to be a biologically active product. Consequently, manufacturers are requested to follow guidelines applicable to biological medicinal products [140].

From a quality perspective, following the 2008 contamination crisis involving over-sulphated chondroitin sulphate (OSCS), regulators now advise on the development of highly accurate, specific and sensitive methods to confirm the identity and purity of UFH [141], thereby increasing analytical complexity, associated costs, and time to release batches. The compendial reference methods described in the United States Pharmacopeia (USP) and other major pharmacopeias include high-field (≥ 500 MHz) 1 H nuclear magnetic resonance (NMR) spectroscopy and strong anion exchange high-performance liquid chromatography (SAX-HPLC [142–144]. 500 MHz NMR spectroscopy provides high resolution and discriminatory power, by identifying 16 UFH characteristic peaks [145]. Deviations from the expected peak pattern are indicative of contaminants, degradation products or a suboptimal heparin sample. SAX-HPLC is employed for the separation and detection of potential UFH impurities. Furthermore, size exclusion chromatography is used to characterize the molecular weight distribution profile of UFH samples, reflecting regulatory expectations for batch-to-batch molecular weight consistency. Beyond these compendial assays, a broad range of complementary analytical techniques can be applied to (i) identify and separate impurities, (ii) enhance structural elucidation, and (iii) assess molecular size characteristics [145]. Within the first group, techniques such as polyacrylamide gel electrophoresis (PAGE), Agarose gel electrophoresis, Weak anion exchange (WAX)-HPLC, Reversed-phase ion-pair (RPIP)-HPLC, Hydrophilic interaction chromatography (HILIC), mass spectrometry (MS) and fluorescent detection are key examples. Structural characterization may further be supported by infrared (IR) and Raman spectroscopy, as well as multidimensional NMR techniques such as COSY and TOCSY, circular dichroism (CD), among others. Finally, to evaluate the size of heparin samples, methods such as ultracentrifugation, 13 C NMR and MS, between other, may complement size exclusion chromatography results [145].

From an analytical point of view, the biological activity of all UFH products is standardized internationally according to their anti-coagulant effect. However, this particular effect is not the main one in lung diseases, therefore it is necessary to develop methods able to standardize UFH activity for the pharmacological effects targeted in these diseases, specifically, anti-inflammatory and anti-microbial (anti-viral, anti-bacterial) [1]. Such methods should be applicable to the final product, but also for biological samples, where UFH should be quantified.

There are technological hurdles that should be regarded while developing a heparin-containing formulation for pulmonary delivery. These are mainly related to the device required to generate the aerosol. To ensure a uniform lung deposition, the aerosol must display a defined aerodynamic particle size, compatible with the target area [126]. Moreover, it is mandatory to meet other CMC requirements such as drug product stability, as well as human factor aspects involving applicability, interpatient variability and more [126]. As UFH is derived from a biological source, it inherently displays a higher molecule size and an increased tendency to self-arrange/rearrange, thus making the manufacturing process particularly sensitive and requires careful control. In addition, UFH is susceptible to physical and chemical stresses, such as the shear forces generated during the aerosolization process, as well as temperature fluctuations and exposure to humidity [126].

To better highlight the complexity of such formulations, the following table presents an overview of the pharmaceutical development studies, required by the EMA, that need to be presented while developing such products (Table 5).

**Table 5:** Pharmaceutical development studies required for nebulization products to be presented to EMA

Physical characterization	Single dose product	Multidose product
	Yes	Yes
Minimum fill justification	Yes	Yes
Extractable volume	Yes	No
Extractables / leachables	Yes	Yes
Single-dose fine particle dose	No	No
Aerodynamic particle / droplet size distribution	Yes	Yes
Uniformity of delivered dose and fine particle dose through container life	No	No
Uniformity of delivered dose and fine particle dose over patient flow rate range	No	No
Aerodynamic particle size distribution with spacer use	No	No
Actuator / mouthpiece deposition	No	No
Delivery rate and total delivered dose	Yes	Yes
Shaking requirements	Yes, if suspension	Yes, if suspension
Initial & re-priming requirements	No	No
Cleaning requirements	No	No
Low temperature performance	No	No
Performance after temperature cycling	No	No
Effect of environmental moisture	No	No
Robustness	No	No
Delivery device development	Yes	Yes
Preservative effectiveness / efficacy	Yes, if present	Yes, if present
Compatibility	Yes	Yes
Spray pattern / plume geometry	No	No

**Fig. 2:**
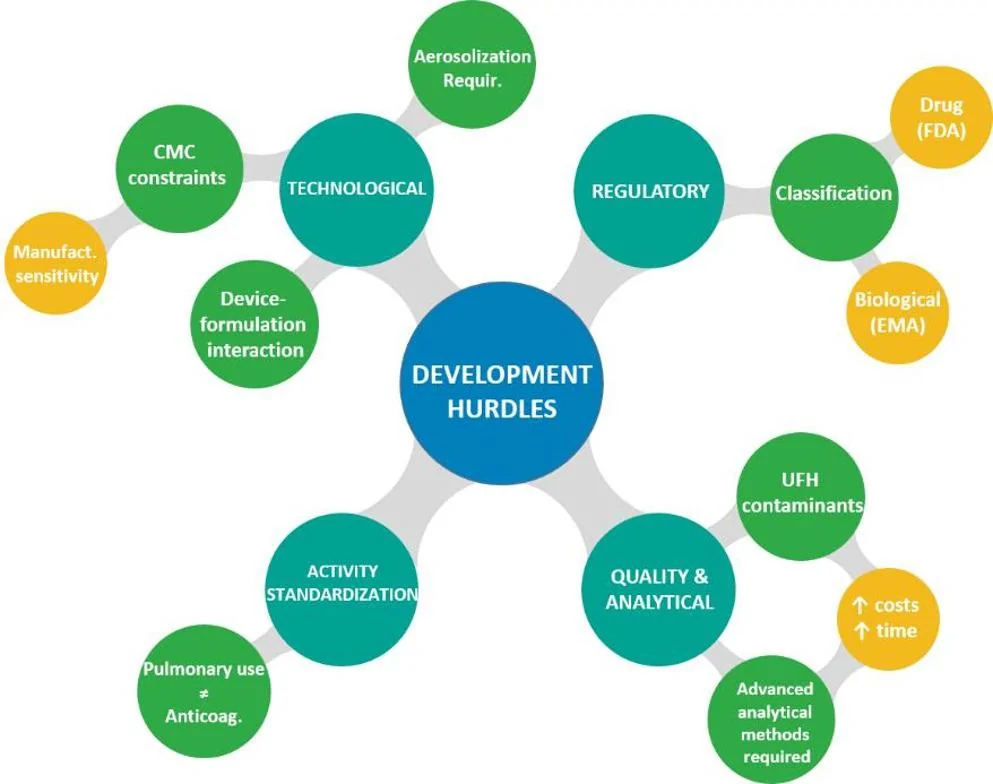
Key hurdles in the pharmaceutical development of nebulized unfractionated UFH formulations targeting lung diseases

## Future of inhaled Heparin

Over the past several decades, a substantial body of clinical evidence has accumulated supporting the safety and efficacy of inhaled heparin across a wide spectrum of respiratory diseases, including acute lung injury, asthma, allergic airway disease, cystic fibrosis, chronic obstructive pulmonary disease, and respiratory infections, most notably COVID-19. During the pandemic, UFH assumed a central role in disease treatment and/or prevention and was classified as a critical life-saving drug [140]. As one of the few pharmaceuticals with a “demand-constant” status, the global heparin market is projected to grow at a compound annual growth rate (CAGR) of 2.7% between 2024 and 2030, increasing from USD 7.56 billion in 2023 to USD 9.03 billion in 2030 [140, 146]. Although much of this growth is related to subcutaneously administered LMWHs, these trends collectively explain why heparin has become a subject of growing interest for both academia and the pharmaceutical industry. However, the pharmaceutical development, and consequently the regulatory approval of a nebulized UFH formulation targeting lung diseases, maybe challenging.

Pulmonary delivery of heparin in the clinic has been investigated mainly using nebulisers. Collectively, these devices have been used to deliver doses ranging from 50,000 to > 400,000 units/day in clinical settings, where UFH has been shown to reach both upper and lower airways effectively. In the meta-trial by van Haren et al. (2025), different nebulisers were used: jet nebulisers (also referred to as Venturi systems and compressed air nebulisers) and vibrating mesh nebulisers [13]. Although the authors noted this variation as a study limitation—since vibrating mesh nebulisers produce finer particles and may deliver more drug to the distal airways—no heterogeneity was found when the results were analyzed together. This indicates that the overall treatment effect was consistent across the studies [13]. In fact, nebulisers enable the off-label use of approved injectable formulations, suitable for ventilated and ICU patients, as well as allowing for flexible dosing. However, the development of formulations specifically tailored for pulmonary delivery would be highly valuable, as this route presents a range of unique characteristics that become particularly relevant in disease states. In addition, the off-label use of UFH presents operational challenges mainly caused by the physicochemical properties of heparin that at therapeutic doses, due to its polyanionic nature often resulting in highly viscous formulations. These limitations underscore the need for innovative formulation strategies to improve aerosol flow, reduce dosing inefficiencies, and enable reliable pulmonary administration. Alternatively to nebulisers, heparin could be delivered using DPI, pMDI, or SMI. However, each of these options has limitations: DPI delivery of heparin is largely confined to research settings, pMDIs face solubility challenges as heparin does not dissolve well in common propellants, and in SMIs, the relatively high viscosity of heparin solutions can reduce delivery efficiency.

In addition to these conventional dosage forms, nowadays there are advanced drug delivery systems being developed to substantially improve the pulmonary delivery of UFH. These include hydrogels and nanocarriers. Nanocarriers represent a cutting-edge approach to drug delivery. They are nanoscale vehicles which are designed to encapsulate, protect and release therapeutic agents in a controlled manner [1, 147]. Due to the large molecular size and negative charge of heparin, with poor oral bioavailability, an advantage of nanocarriers is the possibility to protect heparin from degradation thus enhancing absorption and providing a controlled, sustained release. Nanocarriers can be engineered to target specific tissues or cells, such as inflamed tissues or cancer cells, thereby increasing the therapeutic effect while minimizing side effects. By using heparin-loaded nanocarriers, it may be possible to achieve localized anti-inflammatory effects without significant systemic anticoagulation, which is particularly beneficial in conditions where bleeding risk is a concern. Heparin-loaded nanocarriers have shown promise in enhancing wound healing. For example, hydrogels and nanofibers that release heparin can promote angiogenesis (formation of new blood vessels) and reduce inflammation at the wound site, leading to faster healing. Promisingly, in cancer treatment, heparin’s ability to inhibit metastasis and modulate the tumour microenvironment can be harnessed using nanocarriers. Targeted delivery of heparin to tumour sites can enhance its therapeutic effects while minimizing systemic exposure. As briefly touched upon in this body of work, research is increasingly focusing on the inhalation route for delivering heparin and its derivatives directly to the lungs. Translation of this approach toward clinically viable products is being actively pursued through the development of purpose-designed inhaled UFH formulations [148]. This method could enhance local therapeutic effects while minimizing systemic side effects. Clinical trials are ongoing to evaluate the efficacy and safety of inhaled UFH, LMWH and non-anticoagulant derivatives in managing respiratory diseases.

As research advances, we are optimistic there may be opportunities to tailor heparin therapies based on individual patient profiles, including genetic factors that influence drug metabolism and response. This personalized approach could optimize treatment outcomes and minimize adverse effects. Ongoing research and clinical trials will be crucial in establishing the safety and efficacy of new heparin formulations and delivery methods. The results of these studies will guide clinical practice and inform regulatory decisions regarding the use of heparin in various therapeutic contexts.

## Data Availability

No datasets were generated or analysed during the current study.

## Abbreviations

ACE2: Angiotensin-Converting Enzyme 2
ARDS: Acute Respiratory Distress Syndrome
BLH: Bovine Lung Heparin
BSE: Bovine Spongiform Encephalopathy
CAGR: Compound Annual Growth Rate
CF: Cystic Fibrosis
CMC: Chemistry, Manufacturing and Controls
COP: D Chronic Obstructive Pulmonary Disease
CPAP: Continuous Positive Airway Pressure
DPI: Dry powder inhalers
ECMO: Extracorporeal Membrane Oxygenation
FEV_1_: Forced Vital Capacity
FVC: β-D-glucuronic acid
GlcA: GlcN α-D-glucosamine
HFNC: Flow Nasal Cannula
HFNO: High Flow Nasal Oxygen
HFOV: High Frequency Oscillatory Ventilation
HILIC: Hydrophilic interaction chromatography
ICU: Intensive Care Unit
IdoA: 1,4 linked α-L-iduronic
IR: Infrared
LMWH: Low Molecular Weight Heparin
MDIs: Metered-Dose Inhalers
MMAD: Mass Median Aerodynamic Diameter
MS: Mass spectrometry
NETs: Neutrophil Extracellular Traps
NIV: Non-Invasive Ventilation
NMR: Nuclear Magnetic Resonance
ONS: Official National Statistics
OSCS: Over-Sulfated Chondroitin Sulfate
PAGE: Polyacrylamide GEl electrophoresis
pMDIs: Pressurized Metered-Dose Inhalers
PMH: Porcine Mucosal Heparin
RPIP-HPLC: Reversed-Phase Ion-Pair High-Performance Liquid Chromatography
SAX-HPLC: strong anion exchange high-performance liquid chromatography
SLPI: Secretory Leukocyte Protease Inhibitor
UFH: Unfractionated Heparin
ULMWH: Ultra-low molecular weight Heparin
VHCs: Valved Holding Chambers
WAX-HPLC: Weak anion exchange high-performance liquid chromatography
